# Natural Drug-Loaded Bimetal-Substituted Hydroxyapatite-Polymeric Composite for Osteosarcoma-Affected Bone Repair

**DOI:** 10.3389/fcell.2021.731887

**Published:** 2021-09-20

**Authors:** Yanjun Wang, Yongfeng Yao, Muthupandi Thirumurugan, Selvakani Prabakaran, Mariappan Rajan, Kai Wang

**Affiliations:** ^1^Department of Orthopedics, Daxing Hospital, Xi’an, China; ^2^Biomaterials in Medicinal Chemistry Laboratory, Department of Natural Products Chemistry, School of Chemistry, Madurai Kamaraj University, Madurai, India; ^3^Department of Hematology and Oncology, Honghui Hospital, Xi’an, China; ^4^Department of Physiology and Pathophysiology, Air Force Medical University, Xi’an, China

**Keywords:** biocompatible, hydroxyapatite, new cell development, osteosarcoma, umbelliferone

## Abstract

Repairing segmental bone deformities after resection of dangerous bone tumors is a long-standing clinical issue. The study’s main objective is to synthesize a natural bioactive compound-loaded bimetal-substituted hydroxyapatite (BM-HA)-based composite for bone regeneration. The bimetal (copper and cadmium)-substituted HAs were prepared by the sol-gel method and reinforced with biocompatible polyacrylamide (BM-HA/PAA). Umbelliferone (UMB) drug was added to the BM-HA/PAA composite to enhance anticancer activity further. The composite’s formation was confirmed by various physicochemical investigations, such as FT-IR, XRD, SEM, EDAX, and HR-TEM techniques. The bioactivity was assessed by immersing the sample in simulated body fluid for 1, 3, and 7 days. The zeta potential values of BM-HA/PAA and BM-HA/PAA/UMB are −36.4 mV and −49.4 mV, respectively. The *in vitro* viability of the prepared composites was examined in mesenchymal stem cells (MSCs). It shows the ability of the composite to produce osteogenic bone regeneration without any adverse effects. From the gene expression and PCR results, the final UMB-loaded composite induced osteogenic markers, such as Runx, OCN, and VEFG. The prepared bimetal substituted polyacrylamide reinforced HA composite loaded with UMB drug has the ability for bone repair/regenerations.

## Introduction

Bone is a mineralized connective tissue from which bones, the vertebrate skeleton’s primary segment, are shaped (Frost, 2001). Bones can have several sorts of bone complications, including osteonecrosis, low bone density, osteoporosis, osteogenesis imperfect, Paget’s disease, rickets, bone cancer, etc. (Sabir and Cole, 2019). Bone cancer is the most common bone disease with nearly two thirds of all cancer cases, mainly affecting the younger generation (Marina, 2010). Approximately 1,200 patients are diagnosed with osteosarcoma per year in the United States, and worldwide, there are 3.4 cases per million people in a year (Demellawy et al., 2018; Misaghi et al., 2018). Surgical treatment with bone grafts involve the replacement of defected bone with tissues from the same patient or with artificial or synthetic or natural grafts. Autograft and allograft are two of the treatment methods. This has several limitations: donor site morbidity, restricted bone availability and graft resorption, the risk of disease transmission, restricted bioactivity, and donor dependence (Randal and Betz, 2002). The artificial bone device is widely used for curing bone repair/regeneration because of several advantages, including safety (Reena et al., 2015), resorption (Shi et al., 2021), functionality (Gomes et al., 2019), and osteogenic potential (Mortada and Mortada, 2018). It also overcomes the limitations of other treatments, such as cardiotoxicity, pulmonary toxicity, gradual hearing loss, etc. (Marina, 2010).

Recently, calcium phosphate is widely used for bone growth in the form of hydroxyapatite, calcium diphosphate, calcium triphosphate, octacalcium phosphate, etc. (Lakshmanaperumal et al., 2017). Hydroxyapatite (HA) is an inorganic graft material frequently used for orthopedic applications due to its osteoconduction, hardness, and acceptability by the bone. HA is incredibly closely related to the apatite structure of bone (Hench and Thompson, 2010). However, HA has the advantage over the mineral phase of the natural bone similarity; it also has some limitations for load-bearing applications due to its poor mechanical properties (Kattimani et al., 2016). Besides this, some minerals, such as F, Cl, Na, K, Fe, Zn, Sr, Mg, etc., are substituted to the HA to enhance specific activity, such as antimicrobial, anti-inflammatory, osteogenesis, and angiogenesis (Supova, 2015). Several reports are available because of its enhancing osteogenic nature and reducing bone diseases such as osteosarcoma and osteomyelitis by substituting minerals and biomolecules in HA-based (Lu et al., 2016) and cisplatin-loaded graphene oxide/chitosan/HA composite (Sumathra et al., 2018a,b). The cadmium (II) complex exhibits an anticancer activity against cancer cell lines with IC_50_ of 1.54 ± 0.25 and 31.02 ± 3.76 μmol/dm^–3^ (Huang et al., 2015), and CuO NPs can suppress the proliferation and induce apoptosis of cancer cells (Nagajyothi et al., 2017). Recent studies on HA composites with a combination of organic polymers, such as polylactic acid/HA composite (Blitterswijk and Habibovic, 2014), polylactic acid-HA scaffolds (Zimina et al., 2020), and collagen-HA-based coral (Siswanto et al., 2020), show a significant role in bone regeneration.

The organic phase of bone consists of protein collagen (a polymer), which plays a significant role in its mechanical properties and tissue interactions (Qu et al., 2019; Xiao et al., 2020). Biodegradable polymers, such as poly(lactic acid) (PLA) (Madhavan et al., 2010), poly(lactide-co-glycolide) (PLGA) (Zhao et al., 2021), and chitosan (CS) (Okada et al., 2017) are widely used to develop scaffolds for bone regeneration. Poly(acrylamide) (PAA) is importantly used in biomedical applications (Yang, 2008). Recent studies prove that the polymer PAA and HA mineralized with PAA-dextran hydrogel is a well-applicable composite for bone tissue regeneration (Fang et al., 2019), and **it is reported to have a lead ion removal property due to its porous nature** (Jang et al., 2008).

Single materials cannot fulfill all the properties, especially for the repair and regeneration of bone. Previously, cisplatin, doxorubicin, and gingerol composites were investigated for self-repair of bone implants (Sumathra et al., 2018a,b, 2020). Synthetic anticancer drugs induce toxicity effects against normal cells and low bioavailability. The natural compound Umbelliferone (UMB) is a pharmacologically active agent categorized under the 7-hydroxycoumarin family, and it exhibits various pharmacological activities such as pro-oxidant anti-inflammatory properties against degenerative diseases, microbial infections, and cancer cells in health-related conditions (Mazimba, 2017). UMB was found to exhibit significant anticancer effects via the induction of apoptosis, cell cycle arrest, and DNA fragmentation in cancer cell inhibition (Yu et al., 2015).

Based on these points, here we have prepared for the first time a UMB drug-loaded BM-HA/PAA composite for diseased-affected bone regeneration. The UMB-loaded bimetal-substituted HA was not investigated elsewhere previously for osteosarcoma bone regeneration purposes. The prepared composites were assessed for their physicochemical properties, bioactivity, and biocompatibility by FT-IR, XRD, Zeta potential, SBF immersion, *in vitro* cell studies, and gene expression.

## Experimental Section

### Materials

The following chemicals were utilized and purchased from commercial sources for preparing the composite for osteosarcoma bone repair: Calcium nitrate tetrahydrate (CaH_8_N_2_O_10_), diammonium phosphate [(NH_4_)_2_HPO_4_], cuprous chloride (CuCl), cadmium iodide (CdI_2_), PAA [(C_3_H_5_NO)n], UMB (C_9_H_6_O_3_), and ammonia solution (NH_3_); for preparing simulated body fluid (SBF) solution, the required chemicals, such as sodium chloride (NaCl), sodium bicarbonate (NaHCO_3_), potassium chloride (KCl), dipotassium hydrogen phosphate (K_2_HPO_4_), magnesium chloride (MgCl_2_), calcium chloride (CaCl_2_), 1 M-HCl, sodium sulfate (Na_2_SO_4_), tris buffer, and sodium hydroxide (NaOH) were purchased from Sisco Research Laboratories Pvt., Ltd., Mumbai, India. The solvents used are ethanol (C_2_H_5_OH), and methanol (CH_3_OH), such as received from Sisco Research Laboratories Pvt., Ltd., Mumbai, India. The complete reaction was carried out through double-distilled (DD) water.

### Methods

#### Preparation of BM-HA Ceramic

The sol-gel method was used to synthesize the bimetal-substituted HA (copper and cadmium–substituted HA) (Oliveira Marcela et al., 2003). Briefly, in a 250-mL beaker, 0.4 M of calcium solution was taken, and it was prepared by dissolving the Ca salt in 50 mL of water and stirred using a magnetic stirrer. Then, 0.05 M of cuprous solution and 0.05 M of cadmium solution were added slowly, drop by drop, using burette in calcium solution with minimum 1 h consumption. The solution was maintained at pH 11.0 using ammonia solution. Then, 0.3 M of diammonium phosphate was prepared by dissolving the ammonium phosphate in an aqueous solution and added dropwise to the calcium solution mixture. The solution was left under constant stirring for a day; the solution was filtered, washed with ethanol, and dried in an oven at 75°C. After drying the BM-HA ceramic, it was ground using a blender for uniformity. Then, the BM-HA was sintered in a muffle furnace at 450°C over 5 h to remove impurities before use (Negrila et al., 2018). The HA was prepared separately without substituting the metals following the same procedure.

#### Preparation of BM-HA/PAA/UMB Composite

**SCHEME 1:**
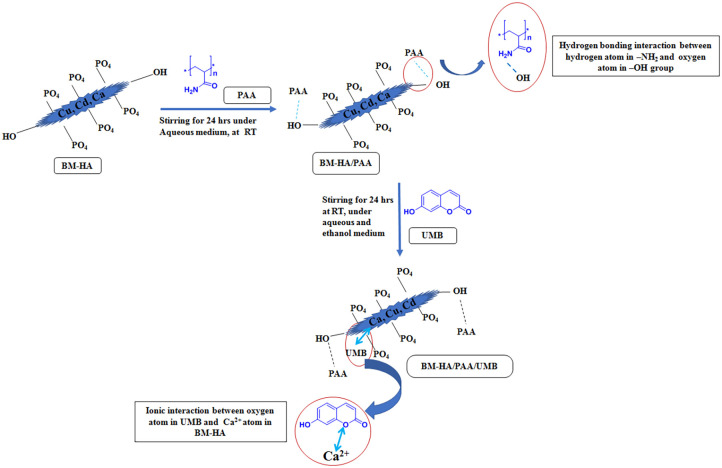
The possible mechanism for the formation of BM-HA/PAA/UMP composite.

Poly(acrylamide) (0.3 g) was dissolved with 25 mL of cold water in a 250-mL beaker and stirred using a magnetic stirrer until it became a jelly solution. Then, 0.5 g of water used by the BM-HA was dispersed in 25 mL of water and added to the above polymeric solution, stirring vigorously until getting a homogenous form. This was followed by 0.3 g of UMB drug in ethanol solution added and stirred over 10 h. The composite solution was allowed to evaporate until all the solvents evaporated. The final composite was scratched and ground by a blender to obtain a fine powdery BM-HA/PAA/UMP composite. Following the above procedure, BM-HA/PAA composite was also prepared without adding UMB drug. Scheme 1 describes the possible mechanism for the formation of each composite as well as the final composite.

### Physiochemical Characterizations

#### FT-IR Analysis

The functional group analysis was carried out for all the prepared composites using the instrument SHIMADZU IRTRACER-100 under the region of wave-number 4000–400 cm^–1^ with 2 cm^–1^ resolutions.

#### XRD Studies

The XRD BRUKER ECO D8 ADVANCE was used to analyze the crystalline nature and phase characteristics for all the composites prepared with the monochromatic Cu Kα source operated at 40 kV and 30 mA. The operating range of the analysis was over 2θ range of 10° to 80° in step scan mode with a step size of 0.02° and a scan rate of 0.02°/min.

#### SEM Studies

The morphology of prepared composites was analyzed with scanning electron microscopy (SEM) combined with energy-dispersive X-ray spectroscopy (EDX) (JEOL JSM- 6400, Japan). SEM images were analyzed by coating the composites in a glass plate by dispersing the composite in ethanol.

#### Transmission Electron Microscopy (TEM) Analysis

The model JEOL JEM 2100 Co., Tokyo, Japan, was utilized for high-resolution transmission electron microscopy (HR-TEM). For TEM analysis, the composite was dispersed in ethanol, and it was coated to the Cu grid for perfect magnification. The SAED pattern was studied to know the crystal structure of the composite.

#### Bioactivity

The SBF was prepared and utilized here due to its similar ionic concentration to human blood plasma. It was prepared by the procedure described in previous literature (Danoux et al., 2014). The final composite was taken and made in pellet form, and it was immersed in a sealed tube. SBF solution was added to immerse the composite and placed at room temperature for 1, 3, and 7 days. For the samples placed for more than a day, the SBF solution was changed daily. After attaining a particular time, the sample was washed and dried. Then, the sample was examined by SEM and XRD analysis to access the bioactivity in terms of apatite formation to make the bonding ability of the composite.

#### Zeta Potential

The zeta potential examination was used to determine the charge of the surface of the composites and the particle size by using the Delsa analyzer instrument (Beckman Coulter) by dispersing the composite in water at room temperature.

### Biological Studies

#### *In vitro* Cell Culture

The human bone marrow mesenchymal stem cells (hBMSCs) purchased from American type culture collection (ATCC PCS-500–012) were maintained in 24-well tissue culture plates with different testing composites, such as BM-HA, BM-HA/PAA, and BM-HA/PAA/UMB, with the medium of normal culture of Dulbecco’s modified eagle medium (DMEM, GIBCO) and with minimal essential media (HiMedia Laboratories) and the supplementation of 10% fetal bovine serum (FBS). To avoid any bacterial infection, drugs, such as penicillin (100 U mL^–1^) and streptomycin (100 U mL^–1^), were given for 48 h. Next to this period, normal media was then refreshed by fresh growth media. Then, the growth circumstance was changed to the humidified atmosphere of 95% air and 5% CO_2_ at 37°C. To remove the unwanted moieties in the composite sample specimens for examinations, they were sterilized at 90°C for 120–150 min in an autoclave and then placed in 96-well cell plates (Pan et al., 2020).

#### Determination of Cell Viability

An MTT assay was carried out to analyze the proliferation of mitochondrial dehydrogenase activity measurements quantitatively. The hBMSC-loaded medium was detached from the culture after 1, 3, 7, and 14 days and shifted into new 96-well cell plates with a density of 1 × 10^4^ cells per well to assess cell proliferation. After 24 h, 2 mL of MTT [3-(4,5-dimethyl-2-thiazolyl)-2,5-diphenyl-2H-tetrazolium bromide] solution in serum-free medium was added to every sample and then incubated under a humidified atmosphere containing 5% CO_2_ for 4 h at 37°C. The solution was removed, and to dissolve the formazan crystals, 10% DMSO was added to every well plate for 15 min. The cell viability was assessed by observing the optical density (OD) values at 570 nm on the spectrophotometric microplate, and the following equation calculated the percentage of cell viability (Wang et al., 2012). Three replicates averaged the experiments.


%ofcellviability=[A]/test[A]×control100

#### Gene Expression Analysis for Cell Differentiation

By utilizing real-time polymerase chain reaction (RT-PCR), mRNA levels were examined to perform the osteogenic differentiation study. After 1, 3, 7, and 14 days culture, the specimens were suspended in 1 mL of cold TRIzol Reagent (Life Technologies Co.) after being cleaned with PBS solution three times. A standard TRIzol protocol was followed to extract the total RNA of each sample, and they were resuspended in 50 μL of RNase-free water. The cDNA was generated using the transcriptase reaction mix (SuperScript III First-Strand Synthesis System, Life Technologies) protocol. The cDNA was stored at −20°C until further studies. The triplicate (*n* = 3) experiments were carried out in quantitative PCR analysis using a Power SYBR Green RT-PCR kit (Life Technologies) protocol. Glyceraldehyde-3-phosphate dehydrogenase (GAPDH) was used as an endogenous housekeeping gene to determine the other gene relative transcripts. Untreated cells were set as control, and the relative marker gene expression levels were designed as one fold. Osteogenic marker genes are osteocalcin (OCN), runt-related transcription factor (RUNx), and vascular endothelial growth factor (VEGF) (Singh et al., 2015).

#### Western Blot Analysis

The concentration of proteins in cell lysates was assessed using Bio-Rad detergent-compatible (Dc) microprotein assay using bovine serum albumin (BSA) as a protein standard. Depending on protein concentration, cell lysates were diluted in RIPA buffer to the gel-loading concentration of proteins (3 μg/μL) mixed with an equal volume of 2x Laemmli buffer and heated for 5 min at 110°C. Protein samples were separated using 10% SDS gel electrophoresis (Bio-Rad, Hercules, CA, United States). The separated proteins were transferred to PVDF membrane Immunoblot (Bio-Rad, United States) employing the wet transfer method. The membrane was then incubated for 1 h at room temperature in a blocking buffer (5% non-fat milk in PBS) followed by incubation with mouse monoclonal IgG primary antibodies (Anti-Osteocalcin, Cat # SC-73464; Anti-VEGF, Cat # SC-7269 from Santa Cruz Biotechnology, India, Anti-GAPDH, Cat # WH0002597M1; and Anti-Runx2, Cat # SAB1412665 from Sigma-Aldrich, India) at 4°C overnight. After the incubation, the membrane was washed three times (5–10 min) with TBS containing 0.1% Tween-20. Immunoblots were detected using anti-mouse horseradish peroxidase–conjugated secondary antibody (goat anti-mouse IgG antibody, Cat # AP308, Sigma-Aldrich, India). GAPDH was used as a loading control. Band intensity was captured using SuperSignal^TM^ West Pico PLUS Chemiluminescent Substrate (Thermo Fisher Scientific, United States) in the ChemiDoc Imaging system (Bio-Rad, United States), and band intensity was measured using the Image J software.

### Statistical Analysis

The obtained triplicated data are given as ± standard deviation. One-way ANOVA built-in origin pro 8.5 was utilized to perform the statistical analysis of the experimental groups. The significance level was set as *p* < 0.05.

## Results and Discussion

### Functional Group Analysis

**FIGURE 1:**
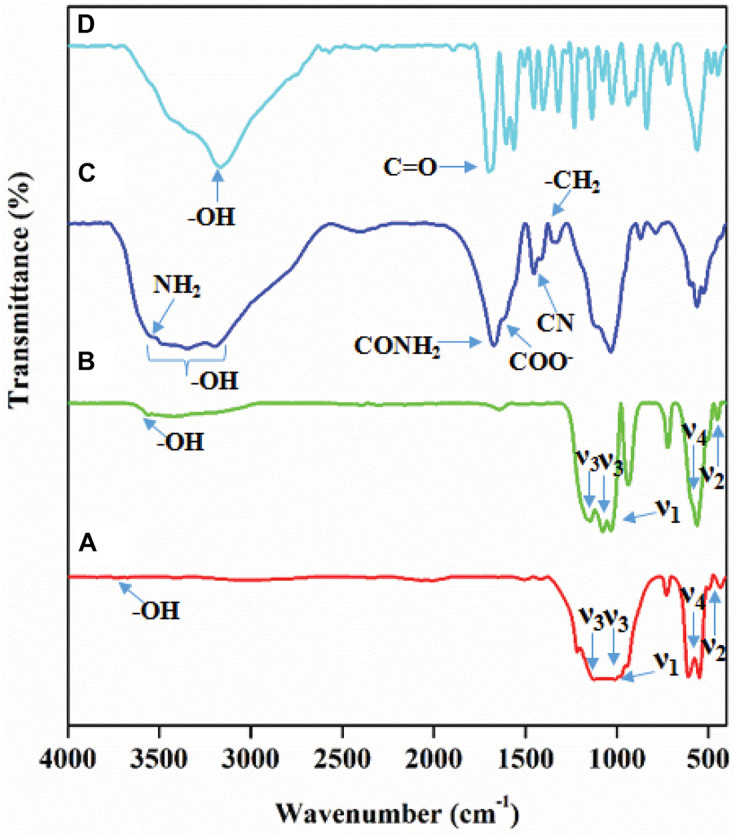
FT-IR spectrum of **(A)** HA, **(B)** BM-HA, **(C)** BM-HA/PAA, and **(D)** BM-HA/PAA/UMB composite.

The functionality of the formed composite was examined by FT-IR spectroscopy, and the results are presented in Figure 1. The FT-IR spectrum given in Figure 1A corresponds to the functional groups of pure HA ceramic. The peaks mainly arise due to the phosphate (PO_4_^3–^) group in the HA lattice. The modes of fundamental vibrations appeared at 1117.63 cm^–1^ and 1026.84 cm^–1^ (ν_3_), 472.63 cm^–1^ (ν_2_), 635.26 cm^–1^ (ν_4_), and 955.0 cm^–1^ (ν_1_) corresponds to the PO_4_^3–^ group in pure HA. The sharp absorption peak appears at 3568.9 cm^–1^ for the hydroxyl group’s vibration mode (-OH) of the HA (Bundela and Bharadwaj, 2012). The Cd and Cu metal substituted in the HA lattice, replacing the Ca^2+^ ions, affected the PO_4_^3^ absorption peak intensity given in BM-HA/PAA (Figure 1B). The FT-IR spectrum clearly showed that the intensity of the ν_2_ absorption peak and an absorption peak of -OH vibrational mode were decreased due to the breakage of the electric charge balance in the HA lattice caused by the substitution of a divalent Ca^2+^ ion with Cu and Cd ions (Arcos and Vallet-Regi, 2020). The positions of the PO_4_^3–^ absorption peaks were retained as placed in the pure HA spectrum. Figure 1C represents the FTIR spectra of PAA polymer, in which the peaks at about 3298, 3184, and 1660 cm^–1^ show the presence of primary amide (-NH stretching vibration) groups of the polymer. Whereas the peaks near 1595 and 1429 cm^–1^ correspond to C = O asymmetric stretching, the strong intensity bands appearing around 3300 and 3200 cm^–1^ are associated with the N-H stretching vibrations due to the stretching of symmetric and asymmetric vibrations of the NH_2_ group. The C-O stretching (amide I) vibrations occur at about 1670 cm^–1^. There is a considerable contribution from C-C stretching and NH_2_ deformation vibrations for this amide I band in PAA. The infrared band observed at 1600 cm^–1^ is due to the NH_2_ bending (amide II band) vibrations. The medium intensity band observed at about 1430 cm^–1^ has been assigned to C-N stretching (amide III), whereas the medium intensity band about 1380 cm^–1^ has been assigned to CH_2_ wagging vibrations (Lili et al., 2012). In the Figure 1D FT-IR spectrum, UMB shows a sharp peak of the -OH group at 3182.55 cm^–1^. It shows C = O stretch at 1726.29 cm^–1^, aromatic C–H stretch from 3061.03 to 3084.18 cm^–1^, and aromatic C = C stretch (Kassim et al., 2013). These peaks may appear due to various functional groups and natural compounds in the UMB compound. These results from FT-IR spectra strongly suggest the formation of desired composites with appropriate interactions.

### Material Phase Analysis

**FIGURE 2:**
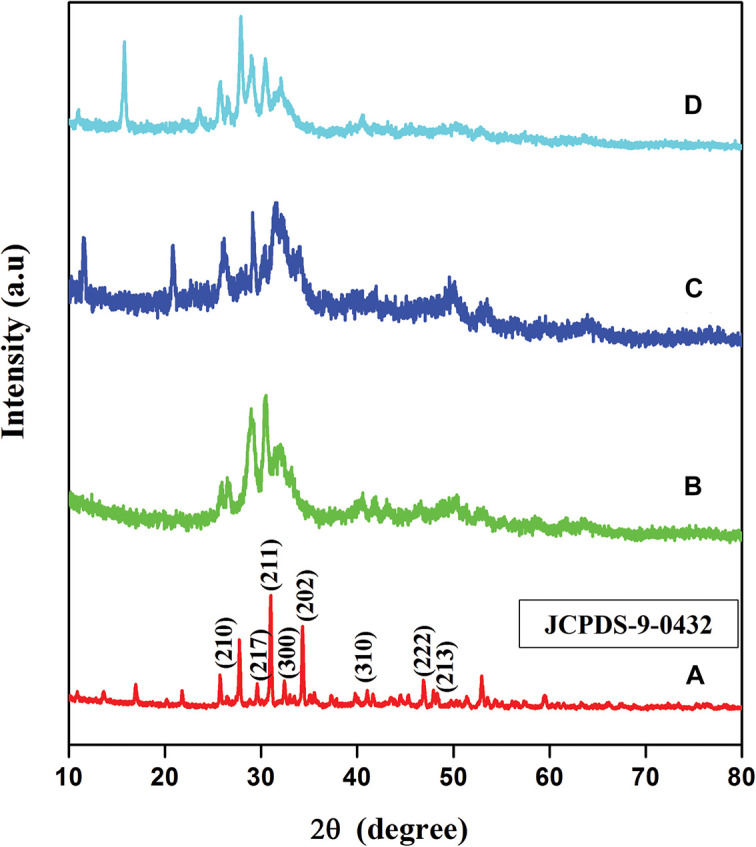
The XRD patterns of **(A)** HA, **(B)** BM-HA, **(C)** BM-HA/PAA, and **(D)** BM-HA/PAA/UMB composite.

XRD examination was used to analyze the crystallinity phase of HA, BM-HA, BM-HA/PAA, and BM-HA/PAA/UMB composites. The XRD patterns of the pure HA ceramic, BM-HA, BM-HA/PAA, and BM-HA/PAA/UMB composites are shown in Figure 2. The diffraction peak in Figure 2A corresponds to pure HA ceramic, which shows the peaks at 2θ values of 25.5°, 29.6°, 31.1°, 32.6°, 34.2°, 39.7°, 47°, and 48.1°correspond to (210), (217), (211), (300), (202), (310), (222), and (213) planes, respectively. The observed diffraction peaks are identified by the preferred JCPDS (File no. 09-0432) report and argue the tremendously crystalline structure of HA (Alexopoulou et al., 2015). The XRD spectrum of BM-HA ceramic corresponds to the peak in Figure 2B. A slight deviation in plane positions occurs, and the decrease in the crystalline nature was noticed when the cadmium and copper were substituted in the Ca^2+^ ion’s matrix. The substitution of Cu and Cd in the HA ceramic affects the crystalline nature of HA. It may be due to the difference between the ionic radius of bimetals and Ca^2+^ as showed by Lakshmanaperumal et al. (2021). Some peaks in the plane of (210), (217), (211), (300), (202), (310), (222), and (213) were deviated from their original position, and the intensity of existing peaks is decreased and broadened during the bimetal substitution. Figure 2C shows the composite with the slight broadening of the apatite peaks, thus suggesting a decrease in HA’s crystallinity due to incorporating amorphous polymer into the crystalline material. Besides this, the spectrum shows a diffraction peak at about 31.9°, indicating well crystalline nature HA even in the composite state. The peak in Figure 2D corresponds to the BM-HA/PAA/UMB composite. The UMB load in the composite shows the intense sharp diffraction peaks of crystallinity at a diffraction angle (2θ) of 15.8°. This confirms the presence of the UMB in the composites. The crystallinity of the composite was altered by adding external organic moiety into the apatite lattice.

### SEM Analysis

**FIGURE 3:**
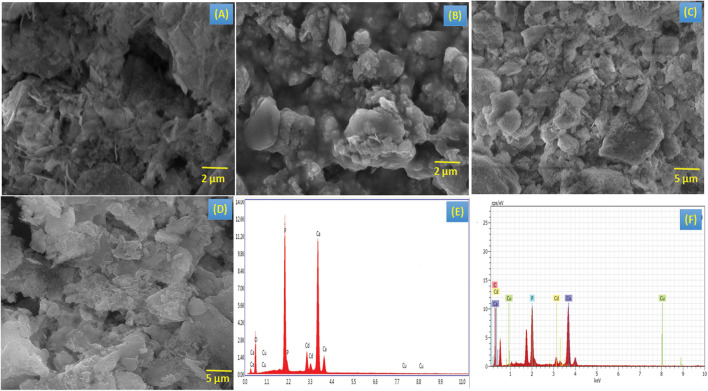
SEM images of **(A)** HA, **(B)** BM-HA, **(C)** BM-HA/PAA, and **(D)** BM-HA/PAA/UMB composites and EDAX images of **(E)** BM-HA ceramic, and **(F)** BM-HA/PAA/UMB composite.

The microstructural examination of the composite HA, BM-HA, BM-HA/PAA, and BM-HA/PAA/UMB was carried out, and the results are given in Figure 3. The SEM morphological features were observed on the compounds of HA, BM-HA, BM-HA/PAA, and BM-HA/PAA/UMB composites as shown in Figures 3A–D. The sharp bed-like morphology was observed for HA ceramic (Figure 3A). After adding minerals, such as Cu and Cd, its morphology was changed into some agglomerated sphere-like particles (Figure 3B). These sphere-like particles will disappear, and the formation of different non-uniform-like morphology appeared after making a composite with a PAA polymer (Figure 3C). The addition of the UMB natural drug makes a sheet and flake-like morphology as shown in Figure 3D. These different morphologies corresponding to different composites show the considerable interaction between the components present in individual composites. The elements in the prepared composite were shown in EDX spectra for BM-HA, and the BM-HA/PAA/UMB composite is given in Figures 3E,F, respectively. The EDX analysis confirmed that the substituted minerals (Cu and Cd) were present as well as the Ca, P, and C elements. The HA was formed with the atomic ratio of 1.73 and 1.87 in the BM-HA and BM-HA/PAA/UMB composite. The obtained atomic percentage of each element from the mapping analysis for the final composite BM-HA/PAA/UMB is given as follows: C (PAA and UMB) = 6%, Ca = 46%, P = 40%, Cd = 7%, and Cu = 1%. The mapping images are shown in Supplementary Figure 1 for the BM-HA/PAA/UMB composite and each element present in this composite. From the elemental mapping of the composite, we can see the equal and uniform distribution of particular elements in the UMB-loaded composite.

### TEM Analysis

**FIGURE 4:**
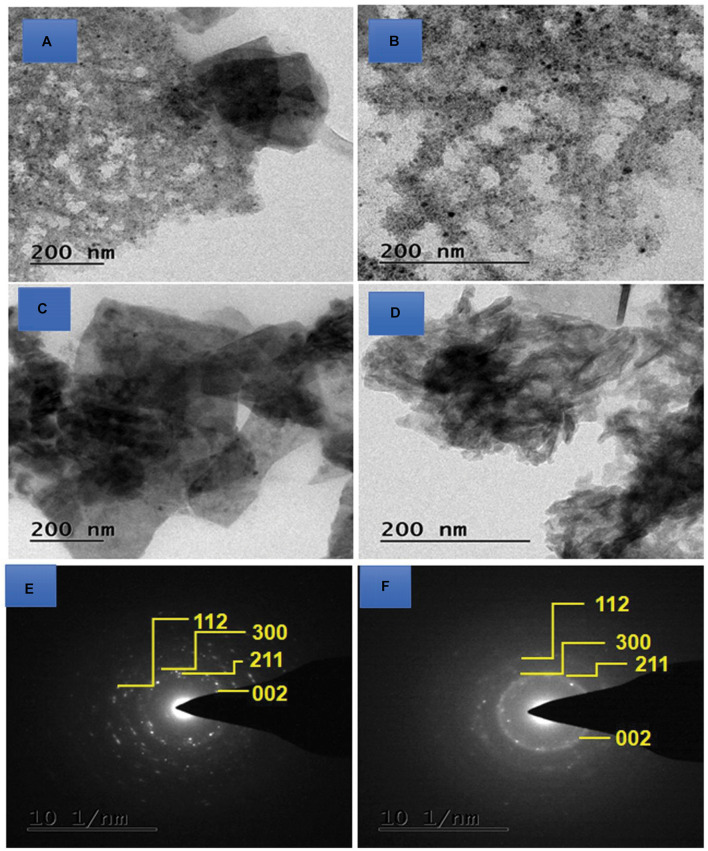
TEM images of **(A)** HA, **(B)** BM-HA, **(C)** BM-HA/PAA, and **(D)** BM-HA/PAA/UMB composites and the SAED pattern of BM-HA/PAA **(E)** and BM-HA/PAA/UMB **(F)** composites.

In addition to SEM observation, the microstructures of the HA, BM-HA, BM-HA/PAA, and BM-HA/PAA/UMB composites were further evaluated with HR-TEM analysis. At the 200-nm level, both HA (Figure 4A) and BM-HA (Figure 4B) show a particle-like morphology with a loosely bound manner. After the polymer and drug addition, the two composites are not positioned compactly. A separate view of BM-HA and the polymer in the BM-HA/PAA composite is in Figure 4C. After the addition of UMB to that composite, some rod-like needles appeared (Figure 4D). We can confirm that the addition of UMB can produce some crystal growth due to the interaction of various functional groups and BM-HA particles. The SAED pattern of the BM-HA/PAA and BM-HA/PAA/UMB composites is shown in the insets of Figures 4C,D. The SAED results firmly stand for the XRD concept of the influence of the HA-based composite in the crystallinity of the BM-HA/PAA/UMB matrix (Figures 4E,F). In both composites, the HA crystalline nature was retained, and a fine crystalline pattern was observed in the BM-HA/PAA/UMB matrix; it was well correlated with the XRD results.

### Surface Charge of the Composites

The zeta potential value was measured in terms of the composite’s surface charge; it is used to determine the implants’ bioactivity. The high value of the zeta potential was observed for the BM-HA/PAA/UMB composite compared with the BM-HA/PAA composite. This increment in zeta potential value may have resulted from the UMB incorporation in the BM-HA/PAA composite because UMB has some negative functional groups. The zeta potential values BM-HA/PAA and BM-HA/PAA/UMB composites are −36.4 mV and −49.4 mV, respectively. We confirm that BM-HA/PAA/UMB serves as a better bioactive implant (Ohgaki et al., 2001). The zeta potential plots for BM-HA/PAA and BM-HA/PAA/UMB are given in Supplementary Figures 2A,B. The electronegative charge-containing surface is a favorable site for the growth of the apatite layer because, initially, the absorption of Ca^2+^ ions is more preferential for that negatively charged surface. As a result of these Ca^2+^ ions, the negative charge decreases and moves toward the positive charge. The absorption of phosphate ions takes place on the positively charged Ca^2+^ surface. Based on the mechanism, the apatite nucleation occurred on the more negatively charged surface of the material (Prabakaran et al., 2021). Supplementary Figures 2C,D show the particle size distribution of the BM-HA/PAA and BM-HA/PAA/UMB composites. The particle size of BM-HA/PAA and BM-HA/PAA/UMB were observed as ∼351.6 and ∼274.1 nm. These can analyze the insertion of UMB in BM-HA/PAA composite decreasing the particle size. It is due to the good interactions of the composite and UMB drug.

### Bioactivity of the Composites in SBF Immersion

**FIGURE 5:**
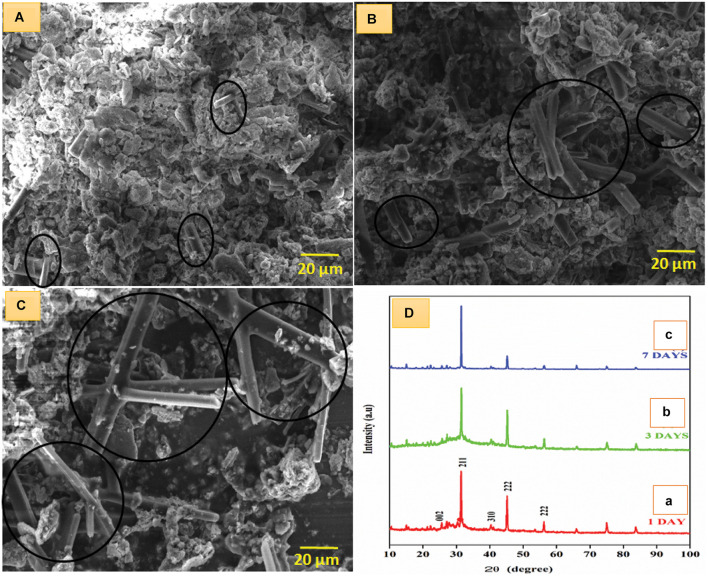
SEM image of apatite formation on SBF immersion at **(A)** 1, **(B)** 3, and **(C)** 7 days and **(D)** XRD pattern of BM-HA/PAA/UMB composite in SBF immersion at **(a)** 1, **(b)** 3, and **(c)** 7 days. The black-colored circle indicates the formation of rod-like apatite particles.

The bone-bonding activity of the prepared composite was the measure of examining its bioactive nature when exposed to *in vivo* implantation. The appearance of apatite crystals act as the bridge between the composite and the host bone tissue. The apatite makes the bio-active bond between the composite and living bone tissue when implanted. It develops the osseointegration of the implant material for new bone formation. The BM-HA/PAA/UMB composite was immersed in SBF solution for 1, 3, and 7 days and the bone-bonding nature examined to find the apatite crystal formation. The morphology of the apatite formation was viewed by SEM analysis and given in Figures 5A–C. As the time of immersion increases, the structure of apatite crystals is more on the BM-HA/PAA/UMB composite. The original morphology disappeared slowly when immersed in SBF for long days due to the formation of apatite crystals as rod-like particles. Figures 5A–C indicate the formation of apatite crystals as small to long rods on the BM-HA/PAA/UMB composite immersed in the SBF solution for 1, 3, and 7 days.

After completing 7 days of immersion, the apatite crystals were formed as a long rod-like structure coated with white-colored HA particles. In all 3 days of immersion, the apatite formation was indicated as a black-colored circle. On the first day of immersion, the rod length was found as ∼5.6 μm, whereas the rod length on the third and seventh days of immersion were found as ∼17 and ∼25.4 μm, respectively, using image J software. The result confirms the bioactive nature of the implant, and it is biocompatible for further *in vivo* implantation in terms of the formation of a bond between the implant and the native bone of the body (Francesco Baino and Seiji Yamaguchi., 2020). Moreover, the formed apatite crystals existed in an exact crystalline nature, evidenced by the XRD diffraction pattern after the BM-HA/PAA/UMB composite was immersed in the SBF solution. After the first and third day of immersion, the peaks in Figures 5Da,b and were raised due to the apatite formation on the BM-HA/PAA/UMB composite. The intense peak at 2θ∼ 28° becomes more intense on the seventh day of immersion as shown in Figure 5Dc. An increase of HA peak at 31.9° in Figure 5Dc shows that the composite’s apatite structure was significantly formed.

### *In vitro* Biological Activity

#### Cell Viability

**FIGURE 6:**
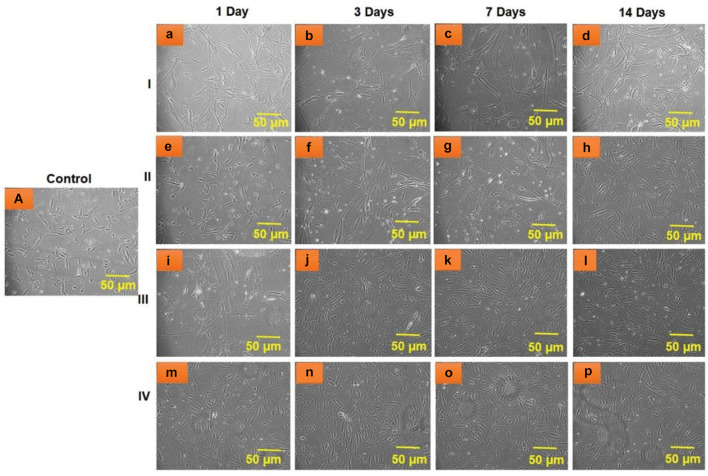
Optical microscopic images of hBMSC viability after treatment with **(I)** HA **(a–d)**, **(II)** BM-HA **(e–h)**, **(III)** BM-HA/PAA **(i–l)**, and **(IV)** BM-HA/PAA/UMB **(m–p)** for various days, such as 1, 3, 7, and 14 days. **(A)** Untreated control cells.

The biocompatibility of the composites, including HA, BM-HA, BM-HA/PAA, and BM-HA/PAA/UMB, were examined *in vitro* by culturing the hBMSCs on each composite separately. In all cases, untreated cells are taken as control cells throughout the examination. Figure 6 shows the optical microscopy images of hBMSCs after treating all composites at various (1, 3, 7, and 14 days) periods. More cells with dense manner were proliferated at 14 days of treatment with the BM-HA/PAA/UMB composite than other composites. Moreover, the number of dead cells was lower in this case against all other cases.

**FIGURE 7:**
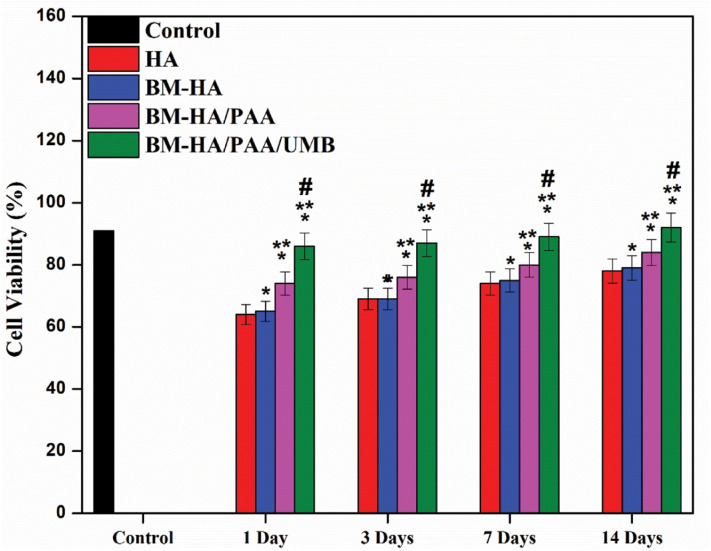
The hBMSC viability on different composites *Comparison of the indicated group with control cells within the same set; *p* < 0.05. ^#^Comparison of the indicated group with HA composite within the same set; *p* < 0.05. **Comparison of the indicated group with BM-HA composite within the same set; *p* < 0.05.

The effect of different composites on the viability of cells started immediately on the first day of the incubation treatment. On the first day of the treatment of hBMSCs cells with varying testing samples (HA, BM-HA, BM-HA/PAA, and BM-HA/PAA/UMB), the viability of cells was greatly affected (Figure 7). The addition of extra components (bimetals, PAA, and UMB) into HA ceramic also involves cell proliferation at all periods, including the first day of treatment. In detail, the viability of cells at all days of treatment with the BM-HA/PAA/UMB composite was significantly higher than control cells and the other three composites (Figure 7). The highest cell viability occurred at 14 days of treatment with BM-HA/PAA/UMB composite (92%). For the osteoblast activity of UMBs, BM-HA/PAA composite shows higher cell growth. A significant difference between the viability of cells on BM-HA/PAA and BM-HA/PAA/UMB composites was observed. From these results, the prepared BM-HA/PAA/UMB composite has a higher ability of stem cell proliferation than other composites due to the presence of all components. The UMB drug does not negatively affect the viability of hBMSCs and helps in the proliferation of the hBMSCs.

#### Osteogenic Differentiation Assessment by Gene Expression and Protein Analysis

**FIGURE 8:**
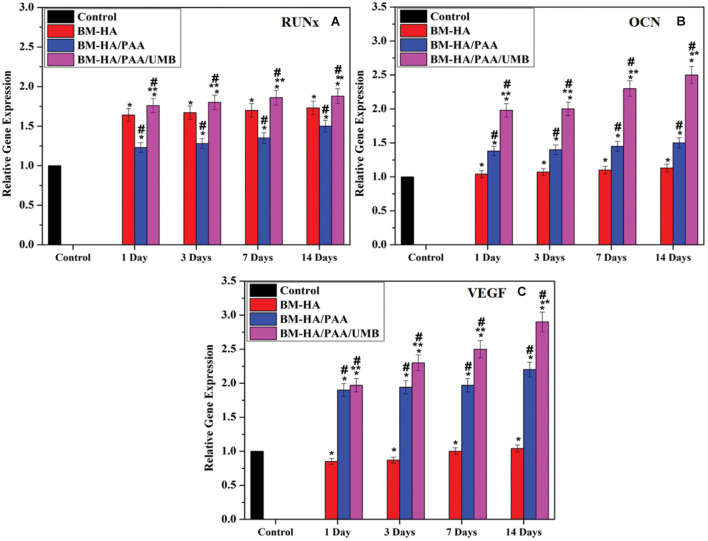
Specific osteogenic differentiation markers **(A)** Runx2, **(B)** OCN, and **(C)** VEGF were assayed by qRT-PCR analysis. *Comparison of the indicated group with control cells within the same set; *p* < 0.05. ^#^Comparison of the indicated group with the BM-HA composite within the same set; *p* < 0.05. **Comparison of the indicated group with the BM-HA/PAA composite within the same set; *p* < 0.05.

**FIGURE 9:**
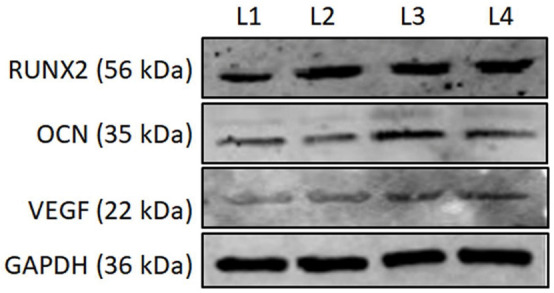
The Western blot images of RUNX2, OCN, and VEGF on the 14th day posttreatment with various composites. L1, Control; L2, BM-HA treated; L3, BM-HA/PPA treated; L4, BM-HA/PPA/UMB treated. GAPDH, Loading control.

For the osteogenic differentiation of hBMSCs in all composites utilized for cell viability analysis, here we have executed the differences of osteoblast gene markers quantitatively by RT-PCR analysis. The mRNA expression of essential osteogenic marker genes, such as RUNx, OCN, and VEGF, were analyzed for 14 days. Quantitative analysis of osteogenic marker gene (RUNx, OCN, and VEGF) expression by the BM-HA, BM-HA/PAA, and BM-HA/PAA/UMB composites is given in Figures 8A–C. The overall expression of the osteogenic marker genes in the cells treated with the pure BM-HA/PAA and M-HA/PAA/UMB composites showed a significant increase in osteogenic marker gene levels than the control cells. Figure 8A shows the RT-PCR analysis of RUNx, and its level has significantly increased as the treatment period increases for all the stages of composites. The comparison between the nanocomposites showed that the relative gene expression of osteogenic marker genes in BM-HA/PAA- and BM-HA-treated cells is lower than BM-HA/PAA/UMB. After adding UMB to the composite, the relative gene expression was significantly higher than control (untreated cells). It may be due to the ability of UMB to suppress the RANKL-induced Akt-c-Fos-NFATc1 signaling pathway and the attenuation of osteoclast-specific genes, such as TRAP, OSCAR, ATP6v0d2, and CtsK (Sung Chul et al., 2019). Another specific OCN marker is a late-stage osteogenesis and mineralization marker; the prepared composite evaluated the OCN expression, and the results are given in Figure 8B. With increasing treatment time, the expression level of the OCN gene was elevated, suggesting osteogenic differentiation of hBMSCs on the BM-HA/PAA/UMB composite. The expression levels of the OCN gene treated with the M-HA/PAA/UMB composite were significantly higher than those with the BM-HA and BM-HA/PAA composites. Like OCN, the relative gene expression of VEGF is increased substantially in the BM-HA, BM-HA/PAA, BM-HA/PAA/UMB composites than in the control (Figure 8C). The BM-HA/PAA/UMB composite cells showed a significant increase in the levels of all three osteogenic marker genes on day 14 of incubation compared with the other composites and other incubation days, indicating this gene expression is time-dependent. In particular, the VEGF gene—an important gene for osteogenesis—expression levels were more than the other genes. Similarly, the Western blot analysis (Figure 9) reveals an increase in the levels of osteogenic marker proteins (Runx2, OCN, and VEGF) in the BM-HA/PAA/UMB-treated group than the control. These results reveal the stem cell proliferation and differentiation capability of the BM-HA/PAA/UMB composite. Among the various composites, the UMB-incorporated composite has significantly higher expression among other composites. The UMB drug loading does not depress the bone cells or relative genes as shown by Sung Chul et al. (2019). The osteogenic ability of the BM-HAP was not affected by the loading of the UMB drug.

## Conclusion

The natural bioactive compound UMB successfully loaded into the PAA-reinforced Cu- and Cd-substituted HA matrix. The FT-IR and XRD expressed the purity and expected crystallinity phases of the synthesized composites. The morphology of the composites was confirmed by SEM and TEM observation. The zeta potential value of the final composite is −49.4 mV, which is lower and more negative than the BM-HA/PAA composite (−36.4 mV). The anticancer drug was released from the composite in a controlled manner and analyzed. *In vitro* experiments in hBMSC viability reveal that the prepared BM-HA/PAA/UMB composite shows more bioactive osteogenic potential among other groups, such as BM-HA and BM-HA/PAA composites. Thus, the synergetic osteogenic ability of Cu and Cd ions and organic UMB compounds in the BM-HA/PAA/UMB composite was clearly evaluated. Therefore, our work expresses that the natural UMB compound enhances the osteoblast cell growth when combined with the BM-HA/PAA composite. It may be used for disease-affected bone regenerations after *in vivo* and clinical evaluations.

## Data Availability Statement

The original contributions presented in the study are included in the article/Supplementary Material, further inquiries can be directed to the corresponding author.

## Author Contributions

YW, YY, MT, and SP: conceptualization, methodology, software, formal analysis, investigation, data curation, writing original draft preparation, and visualization. KW and MR: conceptualization, methodology, software, validation, formal analysis, investigation, resources, data curation, writing – review and editing, visualization, supervision, project administration, and funding acquisition. All authors contributed to the article and approved the submitted version.

## Conflict of Interest

The authors declare that the research was conducted in the absence of any commercial or financial relationships that could be construed as a potential conflict of interest.

## Publisher’s Note

All claims expressed in this article are solely those of the authors and do not necessarily represent those of their affiliated organizations, or those of the publisher, the editors and the reviewers. Any product that may be evaluated in this article, or claim that may be made by its manufacturer, is not guaranteed or endorsed by the publisher.
