# Supplementary material for: Natural Drug-Loaded Bimetal-Substituted Hydroxyapatite-Polymeric Composite for Osteosarcoma-Affected Bone Repair
Source: Front Cell Dev Biol. 2021 Sep 20;9:731887. doi: 10.3389/fcell.2021.731887 (PMC8488211; doi:10.3389/fcell.2021.731887)
Supplement: Supplementary file 1 [file Data_Sheet_1.docx]

**Natural drugs loaded Bimetal Substituted Hydroxyapatite–Polymeric composite for Osteosarcoma affected Bone repair**

**Yanjun Wang^a^, YongfengYao^a^, Muthupandi Thirumurugan^b^, Selvakani Prabakaran^b^, Mariappan Rajan^b^, Kai Wang^cd*^**

^a^Department of Orthopedics, Daxing Hospital, Xi'an 710016, China.

^b^ Biomaterials in Medicinal Chemistry Laboratory, Department of Natural Products Chemistry, School of Chemistry, Madurai Kamaraj University, Madurai-625021, India

^c^ Department of Hematology and Oncology, Honghui Hospital, Xi’an, 710068, China.

^d^ Department of Physiology and Pathophysiology, Air Force Medical University, Xi’an, 710032, China.

**Corresponding author:** Kai Wang, Department of Hematology and Oncology, Honghui Hospital, Xi’an, 710068, China; Department of Physiology and Pathophysiology, Air Force Medical University, Xi’an, 710032, China. Email ID: wangkaiceleron@sina.cn

Methods

**Loading capacity analysis**

The loading capacity of the composite was calculated through UV-visible spectroscopic analysis (SHIMADZU UV-1800). The loading capacity was calculated by the formula given below [38]. The UMB drug about 0.4 mg in 5 mL of DD water was added to 1 g of the carrier solution. Then, this mixture was vortexed for 10 min. The vortexed BM-HA/PAA/UBM solution was then centrifuged at 1500 rpm for 30 min. The obtained supernatant of the drug-loaded carrier was separated, and the free UMB molecule was determined by the UV-Visible spectroscopic method at λmax value of 340 nm, using a spectrophotometer (UV 1800, Shimadzu, India).

$$LC=\frac{Total amount of drug-Free amount of drug}{Weight of Dried nanoparticles} \times100$$

***In-vitro*** UMB **release**

The *in-vitro* UMB releasing property of the BM-HA/PAA/UMB composite was studied by the dialysis method in a phosphate solution pH 7.4 for 24 h. At a proper time interval, the drug-released medium was collected. The same amount of fresh medium was refilled. The UV-Visible spectroscopic method determined the free UMB molecule at λmax value of 340 nm, using a spectrophotometer (UV 1800, Shimadzu, India). The process was made with stirring with a magnetic stirrer throughout the assessment. At last, the percentage of drug release was determined by the formula

Drug release (%) = AR /AC*100

Here,

AR- Absorbance of UMP drug released from the composite

AC - Total quantity of UMP drug loaded onto the composite.

**Real-Time PCR**

Quantitative gene expression was evaluated by SYBR green master mix (Cat # A25741, ThermoFisher Scientific, USA). RNeasy Mini kit (Cat # 74134, Qiagen, USA) was used to isolate RNA from cultured cells according to the manufacturer’s instructions. About 1-2 ug of RNA was used to produce cDNA using the iScript cDNA synthesis kit based on the manufacturer’s protocol (Cat# 1708890, Biorad, USA). The PCR amplification was performed in 40 cycles using the following program: 95 °C for 10 min (hold), 95 °C for 15 s, and 60 °C for 1 min. GAPDH was used as a housekeeping control. Data were analyzed using the 2−ΔΔCt method. The sequences of the primers used are listed in Table 1.

Table 1. The sequences of the primers

| **Gene** | **Forward Primer Sequences** | **Reverse Primer Sequences** |
| --- | --- | --- |
| Runx2 | CCACCGAGACCAACAGAGTC | GTCACTGTGCTGAAGAGGCT |
| OCN | TGAGACCCTCACACTCCTC | ACCTTTGCTGGACTCTGCAC |
| VEGF | CGCTCGGTGCTGGAATTTG | AGGTAGAGCAGCAAGGCAAG |
| GAPDH | AGGTCGGTGTGAACGGATTTG | GGGGTCGTTGATGGCAACA |

**Results and discussion**


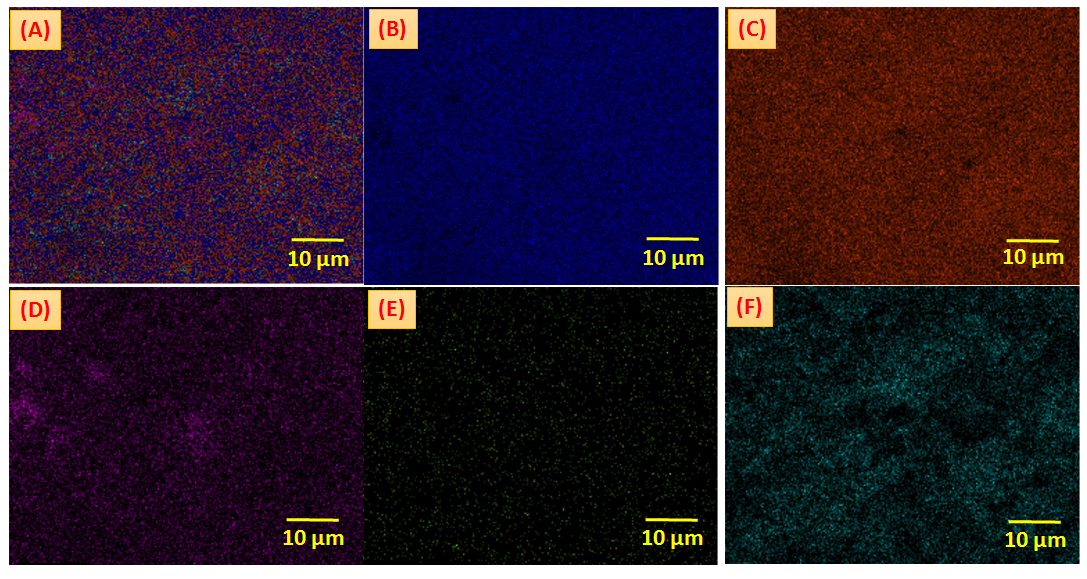


Figure S1. Mapping images of (A) BM-HA/PAA/UMB composite, (B) Ca, (C) P, (D) Cd, (E) Cu, and (F) C.


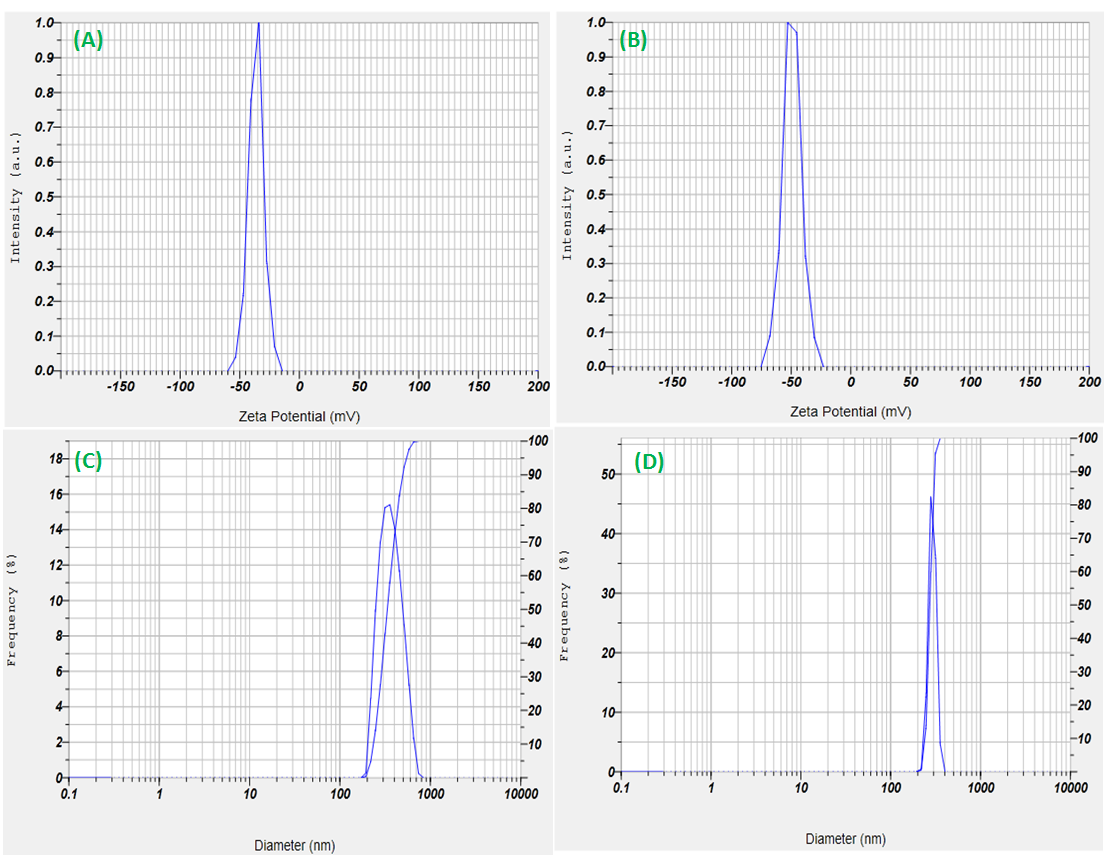
Figure S2. Zeta potential of (A) BM-HA/PAA and (B) BM-HA/PAA/UMB composites and Particle size of (C) BM-HA/PAA and (D) BM-HA/PAA/UMB composites.

**Loading capacity and *In-vitro* drug release**

Examining UMB loading capacity and releasing properties from BM-HA/PAA/UMB composite was investigated through UV-Visible spectroscopy. The BM-HA/PAA/UMB composite was vortexed in 10 min and the UBM loading capacity was observed 57 % (S.Fig 3A). The *in-vitro* drug-releasing studies was carried out at a pH of 7.4. Initially at 0 min, there is no UBM peak absorbance was observed and the time increases the UBM concentration was increased. S.Fig 3 (B) indicates the UBM releasing profile from BM-HA/PAA/UMB composite though UV-Visible spectroscopy. The UMB release was observed 56.0 % over 24 h in the BM-HA/PAA/UMB composite. From the results, we observed the UBM release in sustainable manner and it prolonged release. S.Fig 3 (C) represents the cumulative release of UMB drug from the BM-HA/PAA/UMB composite.


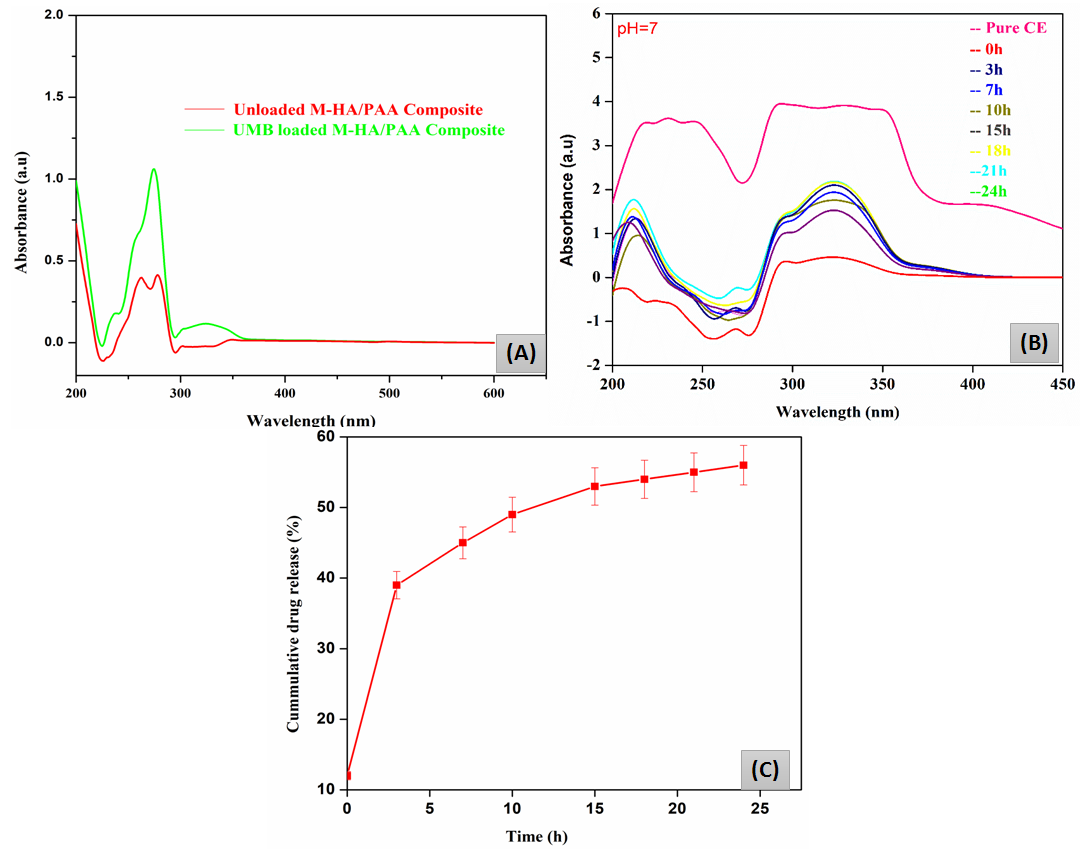


S. Figure 3. Profiles of (A) loading capacity of the composite, (B) drug release and (C) cumulative drug release.
